# Sign and goal tracker rats process differently the incentive salience of a conditioned stimulus

**DOI:** 10.1371/journal.pone.0223109

**Published:** 2019-09-30

**Authors:** Almudena Serrano-Barroso, Juan Pedro Vargas, Estrella Diaz, Patricio O’Donnell, Juan Carlos López

**Affiliations:** 1 Departamento de Psicología Experimental, Universidad de Sevilla, Seville, Spain; 2 Translational Research and Experimental Medicine, Takeda Pharmaceuticals, Cambridge, Massachusetts, United States of America; UiT The Arctic University of Norway, NORWAY

## Abstract

Sign and goal tracker animals show different behavioral patterns in response to conditioned stimuli, which may be driven by different neural circuits involved in processing stimuli. Here, we explored whether sign and goal-tracker profiles implicated different brain regions and responses to incentive salience of stimuli. We performed three experiments using male Wistar rats. Experiment 1 showed that lesioning the medial prefrontal cortex increased the prevalence of the goal-tracker phenotype. Experiment 2 assessed the developmental trajectory of the salience incentive attribution to a conditioned stimulus, showing that increased incentive salience of stimuli increased the prevalence of the sign-tracker phenotype in mature, but not preadolescent rats. In experiment 3, the functional impact of the medial prefrontal cortex circuits was analyzed with a latent inhibition procedure. Sign tracker rats showed a reduced latent inhibition to stimuli previously exposed when compared to goal tracker or intermediate rats. The overall results of this study highlight a key role of the medial prefrontal cortex for sign tracking behavior. The expression of sign and goal tracker phenotypes changed after lesion to the medial prefrontal cortex (experiment 1), differed across development (experiment 2), and showed differences in the attentional processes to previously exposed stimuli, as preexposure to CS was ineffective in sign tracker animals (experiment 3). These data indicate that the responses to the incentive salience of stimuli in sign tracker and goal tracker profiles are likely driven by different neural circuitry, with a different role of prefrontal cortical function.

## Introduction

The incentive salience of a conditioned stimulus (CS) helps an animal with predicting the arrival of an unconditioned stimulus (US). Associative learning theories include incentive salience as an important variable [1–3]. However, animals diverge in the way in which they attribute salience to a CS, as observed in autoshaping procedures. These individual differences have allowed the observation of different patterns of behavior related to incentive salience attributed to a CS, termed goal and sign tracking [4].

Goal- and sign-tracker (GT and ST) animals show different relationships with CS. While GT animals show a behavior focused on the goal or approaching the place where the reward will be released, ST animals focused on the cue [5–7]. This classification, despite being simplistic, assumes different behavioral patterns. Flagel et al. [8] showed that animals bred to enhance response (bred High Responders; bHR) display a ST pattern of behavior, and this pattern correlated with lower levels of anxiety than bred Low Responders (bLR, which show a GT profile), higher levels of aggressive behavior, impulsivity and addiction-related traits [8]. In addition, electrochemical studies have shown higher levels of dopamine release in nucleus accumbens (NAc) after CS presentation in ST animals [7]. These differences could impact risk for mental illnesses [9–11]. In a recent paper, Flagel et al. [12] described the different performance of low/high response rats in an autoshaping procedure after cocaine exposure during adolescence, a critical developmental period for brain regions implicated in these behaviors. Cocaine administration had a higher effect on GT animals compared to ST animals, suggesting GT rats are more sensitive to stimulants than ST rats during adolescence. Similarly, we [10] reported that adult ST animals with a neonatal ventral hippocampal lesion (NVHL) showed a lower response to CS than sham animals, indicating a reduced motivational response to CS in this rodent model of altered medial prefrontal cortex (mPFC) developmental trajectory [13]. This fact is interesting since NVHL procedure disrupts the normal dopamine modulation of corticolimbic circuits [14]. In addition, NVHL animals were less sensitive to dopamine agonists such as quinpirole. Thus, GT and ST rats are likely different in the manner their mPFC processes information.

The mesolimbic dopamine system is essential for attribution of incentive value to an US. Additionally, this neurotransmitter is an important target in major brain diseases such as attention deficit hyperactivity disorder (ADHD), schizophrenia, and substance abuse [15–18]. The NAc is an essential part of the mesolimbic system that receives dopaminergic fibers from ventral tegmental area and is modulated by afferences from the hippocampus, amygdala, and prefrontal cortex among other structures [19–23]. The NAc is necessary for attributing incentive salience to reward cues [24], and mPFC modulates dopamine levels in the NAc by means of excitatory glutamatergic projections. It is possible that the mPFC projection to the NAc modulates attribution of incentive salience to a CS, therefore determining the expression of ST behavior. If so, this structure should play out more strongly role in ST animals than GT in autoshaping procedure. In order to analyze several aspects of the mPFC involvement in ST and GT animals, experiment 1 assessed whether mPFC lesions differentially affected ST and GT behaviors. Experiment 2 analyzed the changes in salience incentive attribution to a CS during a critical period of mPFC development. If mPFC was involved in ST/GT behavior, behavioral patterns would be different as mPFC matured. Lastly, experiment 3 tested the functional impact of mPFC circuits by assessing attentional processing in ST and GT rats with latent inhibition of a tone-shock association. Latent inhibition allows us an indirect measuring of attentional processes to future CS and it has been recently related to mPFC function [25].

### Ethics statement

All procedures used in the present study were in compliance with the regulations of the European Community Council Directive 2010/63/EU and following the Spanish regulations (R.D 53/02013) for the use of laboratory animals. An ethical commission of University of Seville and Institutional Animal Care and Use Committee (Sanidad Animal, Junta de Andalucía) supervised and approved all the procedures and all protocols used in this specific study. The code of the supervision report is: 31/08/2016/153.

## Experiment 1

Previous studies have showed that the mPFC is involved in controlled processes and goal-directed behavior [25,26] that would imply an increased attention to a CS. López et al. [10] found a decrease in motivational processes in adult animals with a NVHL, a procedure that impairs adolescent prefrontal cortex maturation. To determine whether the mPFC is involved in salience incentive processing, we studied the effect of mPFC lesions in an autoshaping procedure. Autoshaping describes the orientation-approach movements to a CS that appears prior to the US [27]. If mPFC is involved in incentive salience of a CS, a behavioral change should be observed in ST, but not in GT rats.

### Materials and methods

#### Animals

Fifty adult male Wistar rats (300-350g) were obtained from the Centro de Producción y Experimentación Animal (Universidad de Sevilla). Animals were housed individually in their home boxes with 12:12 hours light/dark cycle, and all tests took place during the cycle's light period. All animals had access to food and water *ad libitum* during the experiment.

#### Surgery

Under deep isoflurane anesthesia (2–5% in air, flow rate 1 l/min, 5% induction; 2% maintenance; McKinley type 2, Everest), rats were placed in a David Kopf stereotaxic instrument. The mPFC was injured following coordinates 4.7; 4.2; 3.7 mm anterior, ±0.6 mm lateral, and 4.0 mm ventral from Paxinos and Watson [28]. This lesion was made by injecting NMDA (1 mg in 0.1 ml phosphate buffered saline, PBS 0.1 M; see [29]) into the brain through a 10 μl Hamilton syringe (Model 1701 RN). The amount of NMDA solution injected at each site was 0.25 μl, at a rate of 0.05 μl/min. The needle was left in place for an additional minute after the infusion to allow the diffusion of the solution into the tissue. No unexpected adverse effects were observed 48 h after the surgery. Sham animals received a similar manipulation, but saline solution was injected instead of the drug.

#### Histological analysis. Assessment of mPFC lesion placement

**Fig 1:**
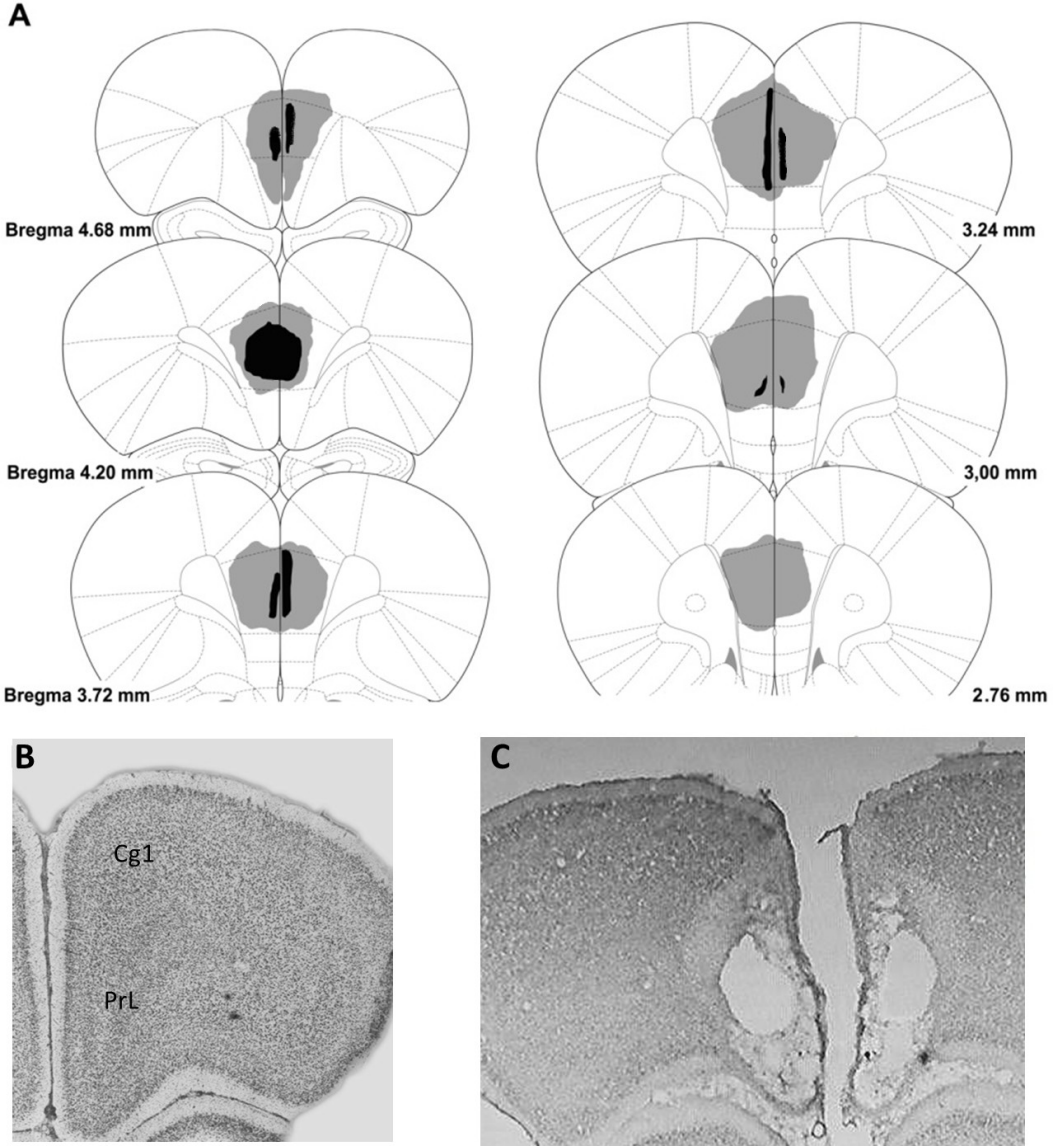
**A. Reconstruction of the mPFC lesions displayed on standard coronal sections from the atlas of Paxinos and Watson [28]**. The largest lesion is shown in pale shading and the smallest in dark shading. **B. Photomicrograph showing a no lesioned coronal brain section. C. A coronal section after excitotoxic lesion of mPFC.** Cg1: cingulate cortex, area 1; PrL: prelimbic cortex.

Upon completion of behavioral testing, rats were deeply anesthetized and perfused transcardially with a fixative solution (10% formalin in phosphate buffer 0.1 M, pH 7.4). The brains were then removed from the skull and placed in 10% formalin and buffered for 3–4 days. Next, the brains were cut with a microtome at the coronal plane at 500020μm thickness oriented according to the atlas of Paxinos & Watson [28] and Nissl stained for histological analysis. Specifically, we stained the tissue with cresyl violet method. This allows to determine the extent of mPFC lesion using an image processing software (ImageJ. NIH, USA, http://rsb.info.nih.gov/ij/). Animals included in this study showed lesions between 49–85% of damage in mPFC (Fig 1), and without significant damage to the adjacent structures.

#### Behavioral testing: Pavlovian conditioning

Four standard boxes from MED Associates (21.1cm x 20.5 cm area, 29.2cm high; Med Associates. St. Albans. VT) were used for Pavlovian Conditioning training. The test chambers were located inside a soundproof test room with constant light and temperature conditions. Methods and procedures were similar to Lopez et al.[10]. Briefly, each chamber was equipped with a feeder located in the middle of the right wall and 3 cm from the bottom grid. The retractable lever was located to the left of the feeder. To increase salience, the lever was illuminated with a white LED. On the opposite wall, at the same height as the feeder there was a monitoring sensor “Nose Poke”. A pellet dispenser provided standard taste pellets into the feeder that served as reinforces. An infrared sensor located inside the receptacle (approximately 1.5 cm from the base of the feeder) recorded each time the animal entered the feeder (when the lever was available). A background white light located on the opposite side to the feeder was on during the whole test. Contacts with the lever were recorded as “Lever Press”; contacts with feeder were recorded as “Nose Feeder”.

Boxes were programmed using Med Associates software. This software recorded the following responses during the whole test: (1) number of lever presses, (2) latency to lever press in the first response for each test, (3) number of feeder entries while illuminated lever was available, (4) latency of the first entry into the feeder when CS was presented, and (5) number of contacts with nose feeder. All test sessions took place between 9:30 and 11:30 a.m. Before the pre-training phase, animals received 5 pellets of the same kind as those used during the test as US in their home boxes for three consecutive days in order to prevent neophobia to the pellets. There were two pre-training sessions during two consecutive days. Thirty pellets were randomly administered for 45 minutes in those training days. The pavlovian conditioning sessions consisted on presenting an illuminated lever (CS) for eight seconds, 25 trials per session. Immediately after CS presentation, the lever retracted and a 45 mg pellet (MLab Rodent Tablet, manufactured by Nottingham University) was provided into the feeder. CS were presented in a 90s variable interval schedule (60s the lowest intertrial interval and 120s the highest). Training sessions continued for four days, allowing classification of rats as ST, GT, or Intermediate (Int) using the Pavlovian Conditioned Approach Index Score (PCA) [30]. This index uses the number of responses to both the lever and the feeder on a rating from -1 to 1. Scores lower than -0.5 indicate a GT phenotype, while ratings higher than 0.5 indicate a ST phenotype. Scores between -0.5 to 0.5 are defined as Int phenotype.

#### Data analysis

In order to analyze the PCA response and nose poke response in sham and mPFC groups, we used parametric T-student test. When it was required, for instance lever press or magazine entries, an ANOVA test (repeated measures) was used including sham and mPFC lesion groups. The different distribution of phenotypes by groups (sham and mPFC) was analyzed with Friedman and Mann-Whitney tests. Significant level was established at p < .05.

### Results

**Fig 2:**
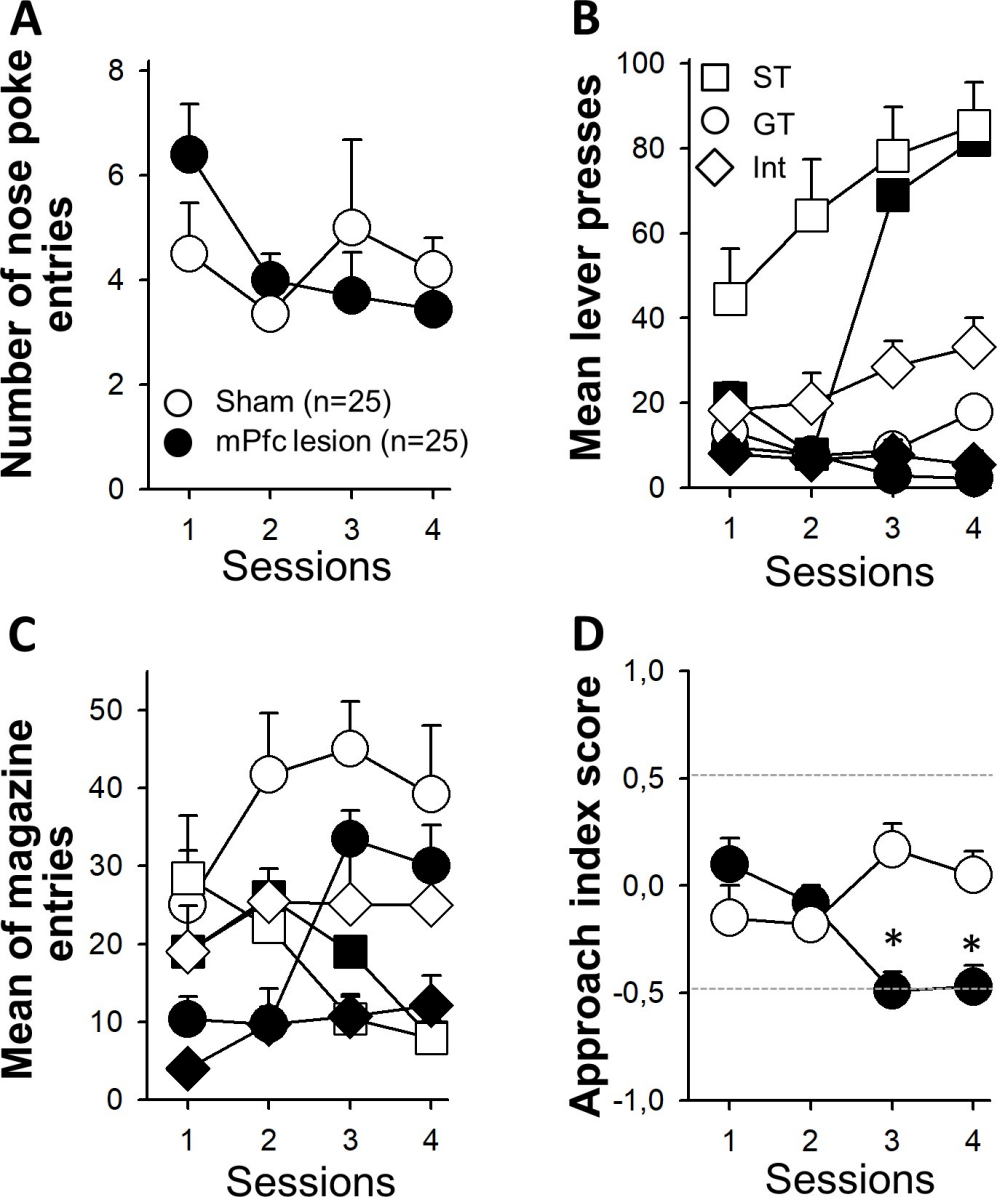
**A. Entries to nose poke device in sham (open circles) and mPFC lesioned (black circles) animals.** The figure includes the mean and standard error from session 1 to 4 for each group including all animals. Dotted lines show the limits between phenotypes. **B. Mean of lever-press behavior and magazine entries (C) when CS was presented** in ST (filled and white squares), GT (filled and white circles), and Int (filled and white diamonds) groups. Filled and open symbols indicate sham or mPFC groups, respectively. **D. Approach Index Score of Sham and mPFC-lesioned rats.** Asterisk indicate significant differences between sham and mPFC lesioned animals.

Rats were trained in autoshaping procedure for four sessions. To evaluate the profiles of sham- and mPFC-lesioned groups, we determine their PCA index. Both groups consumed all pellets in each session, and no motor problems were observed after mPFC lesion. In fact, we did not find differences in the number of nose pokes as a control of animal activity (all T_(48)_<1.4, all p>0.16; Fig 2A). However, a thorough analysis showed that mPFC animals displayed a lower rate of responses in all groups than sham animals. They showed a significant less number of press lever and magazine entries (F(3, 144) = 5,59 and 4,52; p<0.01 for lever press and magazine entries respectively; Fig 2B and 2C).

**Fig 3:**
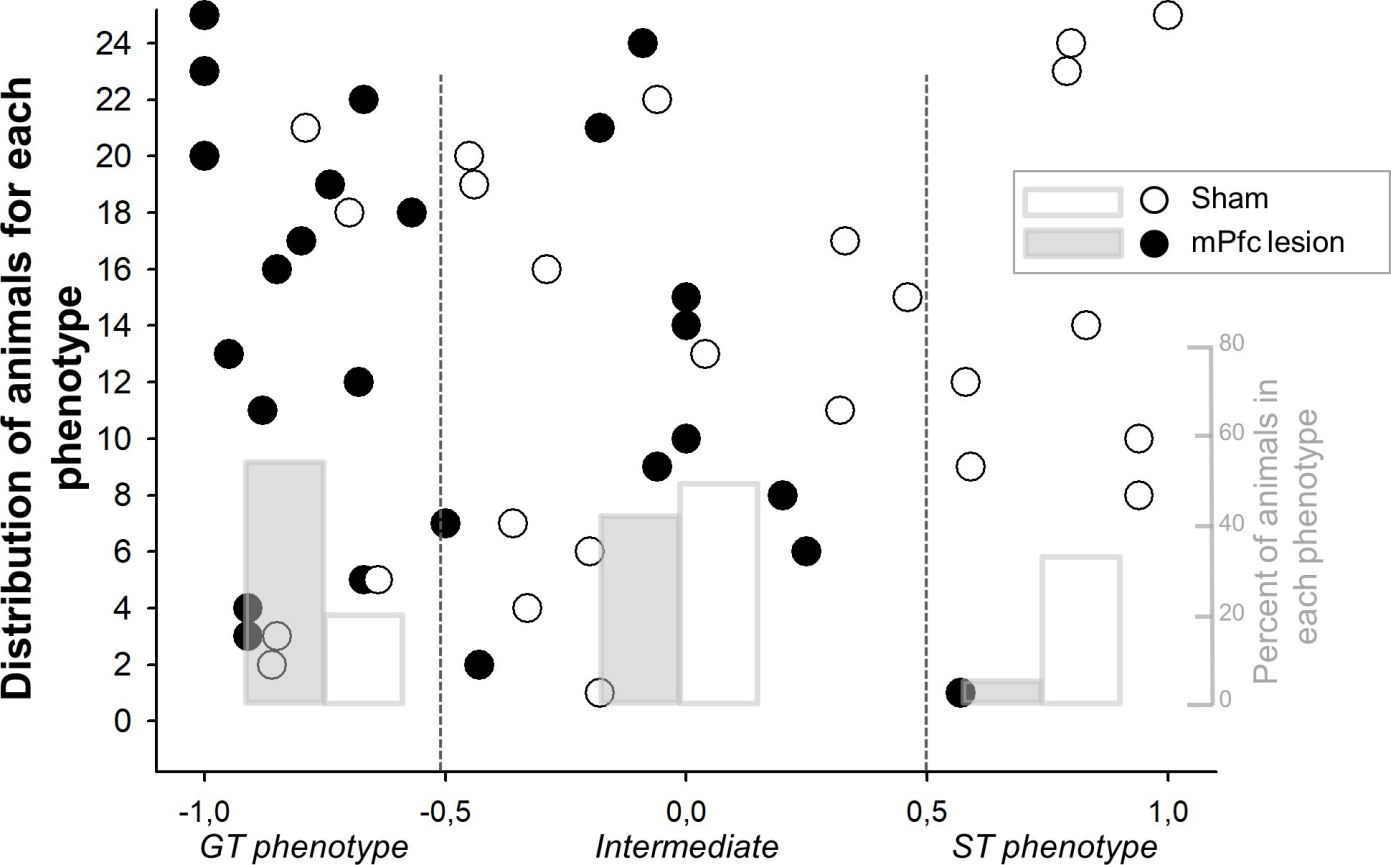
Distribution of GT, ST, or Int phenotypes in sham and mPFC-lesioned animals in session 4. Black and open circles show data from all individual animals. Grey and white bars shows the percent of animals expressing each phenotype.

In order to analyze the global behavior, we used PCA index. This index provided a global measure of lesioned and sham animals performance. We observed a similar activity in PCA for sessions 1 and 2; i.e., the distribution of responses was not different between groups (Mann-Whitney, p = 0.11 and p = 0.30 for sessions 1 and 2; Fig 2D). However, this distribution changed with training. Although sessions 3 and 4 showed similar performances as sessions 1 and 2 in sham rats, mPFC lesioned rats showed a predominant GT behavior (Friedman, p<0.01; Fig 2D), along with a reduced proportion of ST animals (Fig 3; Mann-Whitney, both p<0.01). Sham animals displayed a normalized distribution, where the largest numbers of animals are closer to the mean (Int animals), whereas mPFC-lesioned animals showed a distribution displaced to a GT phenotype (Fig 3).

## Experiment 2

Experiment 1 showed that animals with a mPFC lesion reduced lever-press behavior when CS was available. The maturation of the mPFC during adolescence is a key process for controlling attentional mechanisms [31,32]. If incentive salience of a CS could be related to attentional processes dependent of mPFC maturation, the ST/GT distribution would be different prior to the adolescent critical period compared to a later stage. In order to deepen in this issue, a program of food deprivation was started. Several authors have suggested that some physiological states like hunger could, among other effects, increase incentive salience of food and their associated cues by modulating the motivational value [16,33,34]. This increased motivational could produce an attentional bias towards food-related cue (i.e. [35,36]) and a change in the distribution of ST/GT population. Thus, we analyzed performance in an autoshaping procedure during different motivational states comparing different phases of mPFC maturation, such as pre-adolescent (4 weeks of age) and late adolescent (9 weeks of age) stages.

### Materials and methods

#### Animals

Eighty-four male Wistar rats were used in this experiment. However, twelve of them were removed from the experiment because they did not get the food from the magazine at least 90% of the trials. After the pavlovian conditioning phase, rats were divided in three groups by age. N = 33 (GT n = 7, ST n = 14, Int n = 12) for 4–5 week-old animals (average weight of 150 g; range 130–160 g) and N = 39 (GT n = 7, ST n = 18, Int n = 14) for 8–9 week-old rats (average weight 295 g; range 270–345 g). As in experiment 1, animals were housed individually in their home boxes and all animals had access to food and water ad libitum during first phase of the experiment.

#### Behavioral testing: Pavlovian conditioning

Experiment 2 included three phases of autoshaping procedure. Phase A was a Pavlovian conditioning similar to experiment 1. Phase B included the same Pavlovian conditioning than in Phase 1, but in this case the rats were under a food deprivation schedule. After the initial training in Phase A, rats were deprived of food for three consecutive days until they reached 90% of their weight. Once the targeted weight was reached, Phase B started, consisting on the same pavlovian training received in the previous phase (Phase A), under the same criteria. Deprivation conditions were maintained for the four test sessions and therefore rats were kept at 90% of body weight. After four sessions in Phase B, animal returned to initial conditions and they were trained for four more additional sessions in a Phase A-like (termed Phase A’). Med Pc software recorded the same responses as in experiment 1.

#### Data analysis

Similar to experiment 1, we used an ANOVA test and Tukey post-hoc test. In addition, a repeated measures test helped us to analyze the progressive change across the experiment in all groups.

### Results

**Fig 4:**
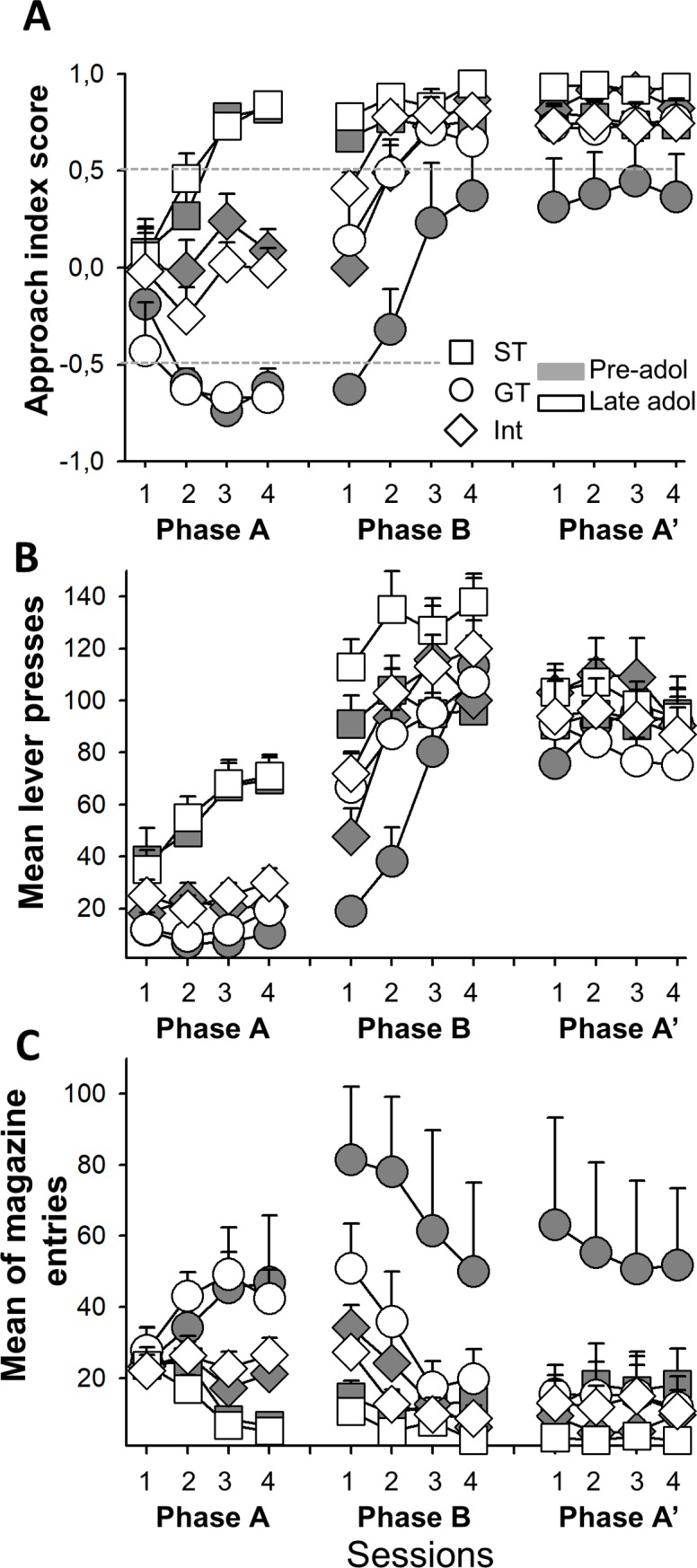
**A. Approach Index Score of pre-adolescent (filled symbols) and Late adolescent (open symbols) groups.** The figure includes mean and standard errors from session 1A to 4A, and 1B to 4B and 1A’ to 4A’ for each group. Dotted lines represent the limits among phenotypes. **B. Mean of lever-press behavior and magazine entries (C) when CS was presented** in ST (filled and open triangles), GT (filled and open circles), and Int (filled and open squares) groups.

As in experiment 1, animals learnt to obtain food from the magazine in the Skinner boxes. The PCA index allowed classifying rats into GT, ST, and Int profiles, depending on their behavior in session 4 (Phase A). A repeated-measures analysis of PCA index revealed a significant interaction of sessions and phenotype in pre-adolescent (F_(6, 90)_ = 4.92; p<0.01; Fig 4A) and late adolescent groups (F_(6, 105)_ = 3.62; p<0.01; Fig 4B) during Phase A. The main effect of session was significant in both GT and ST rats (all p<0.05 for pre-adolescent and late adolescent groups), but not in Int (p>0.3 for pre-adolescent and late Int groups). There was also no differences between pre-adolescents and late-adolescent performance (F(6, 198) = 0.92; p = 0.48).

#### Pre-adolescent group

GT animals increased magazine entries with sessions (F_(3,18)_ = 2.23; p = 0.047), while maintaining constant lever-pressing behavior (F_(3, 18)_ = 0.77; p = 0.52) during Phase A. In contrast, ST animals increased the number of lever presses (F_(3, 39)_ = 5.14; p<0.01) and decreased magazine entries along sessions (F_(3, 39)_ = 7.58; p<0.01). Fig 4B and 4C shows the distribution of responses in both groups in Phase A. Int group maintained the same behavioral pattern for all sessions in Phase A, as no significant differences were recorded (both F_(3, 33)_<0.73; both p>0.53; Fig 4B and 4C).

Once Phase A ended, animals were food deprived to reach 90% of body weight, which took about three days. We then explored the changes in behavior produced by increasing motivation with a food deprivation program (Phase B). ST rats displayed a similar PCA index in Phase A and B (F_(3, 39)_ = 2.33; p = 0.08; Fig 4A), although they increased lever press responses compared to Phase A (repeated measures including Phase A and B F_(7, 91)_ = 6.86; p<0.01; Fig 4A). Magazine entries were similar in Phase A and B (F_(7, 91)_ = 1.85; p = 0.08; Fig 4B). However, GT animals showed a shift in the response to the CS when the motivation increase program started. They increased lever pressing (F_(7, 35)_ = 7.10; p<0.01) and magazine entries (F_(7, 35)_ = 6.86; p<0.01; Fig 4B and 4C respectively) in Phase B compared to Phase A. This increase in both responses suggests a high level of overall activity, shifting their PCA index from GT to Int. Finally, the Int group displayed a displacement in the response ratio, approaching a ST pattern (F_(3, 33)_ = 14.15; p = 0.01; Fig 4A). Thus, increasing incentive salience with food restriction resulted in increased activity but no clear phenotype change in immature rats.

Once finished Phase B animals started Phase A again (Phase A’). We did not find differences between this phase and the last session of Phase B in PCA index (F(3,96) = 1.31; p = 0.27; Fig 4A) in any groups. This is, animals maintained the same profile in Phase B and the new phase A (Phase A’).

#### Late adolescent group

Late adolescent rats performed similar to pre-adolescent rats for all three phenotypes in Phase A. After 4 sessions, we classified the rats as ST, GT or Int. ST rats showed a high number of lever press responses and low number of magazine entries (both F_(3, 48)_>6.18; both p<0.01; Fig 4B and 4C). GT did the opposite, as expected, (F_(3, 18)_ = 3.37; p = 0.041 and F_(3, 18)_ = 1.38; p = 0.28 for magazine and press lever response, respectively; Fig 4B and 4C). Int rats did not show any change across sessions (both F_(3, 39)_>0.61; both p>0.47; Fig 4B and 4C).

Phase B showed a clear difference between pre-adolescent and late adolescent rats for the GT group. While younger animals did not decrease magazine entries, late adolescent rats showed a ST phenotype as they increased lever press responses (F_(7, 42)_ = 24.19; p<0.01; Fig 4B) and reduced magazine entries (F_(7, 42)_ = 4.59; p<0.01; Fig 4C) compared to Phase A. Fig 4A shows the PCA index shift in GT (F_(3, 18)_ = 5.66; p<0.01). In contrast, ST showed a similar phenotype compared to Phase A (F_(3, 51)_ = 4.59; p<0.15) after motivation increase program; that is, high lever press response and low magazine entries response. Int rats showed a similar pattern as pre-adolescent rats, approaching a ST phenotype (F_(3, 39)_ = 12.22; p<0.01; Fig 4A) as evidenced by a lever press increase and a magazine entry decrease (F_(7, 91)_>6.29; both p< 0.01; Fig 4B). Similar to pre-adolescent animals, late adolescent group did not show any shift in the new Phase A (Phase A’) in regard to the end of Phase B (F(3,111) = 0.019; p = 0.97).

We did not find any difference between groups across the experiment for nose poke responses (pre-adolescent Phase A: all F(2,30)<1.64, ps>0.2; Phase B: all F(2,30)<1.13, p>0.33 and Phase A’: all F(2,30)<1.1, ps>0.34; late adolescent Phase A: all F(2,36)<2.11, p>0.13; Phase B: all F(2,36)<2.27, p>0.11 and Phase A’: all F(2,36)<1.6, p>0.21). In this regard, experiment 2 data indicate that under increased incentive salience of stimuli, mature but not preadolescent rats shift towards an ST phenotype.

## Experiment 3

Experiments 1 and 2 have revealed several differences between ST and GT animals, including dependence on intact mPFC, developmental state, and incentive salience state. These profiles could result in a different response to preattentive sensory stimuli, as we recently showed using a prepulse inhibition procedure [10]. In fact, mPFC sends a dense glutamatergic projection to nucleus accumbens, a structure that is itself necessary for LI expression. In addition, several studies have analyzed the involvement of mPFC in LI finding a clear increase of LI after mPFC lesion. Since mPFC showed a different involvement in ST and GT, we wondered if the different behavioral profiles could show any difference in LI processes. With this aim, in experiment 3 we analyzed the attention to a future conditioned stimulus in GT and ST animals using a latent inhibition (LI) procedure.

### Materials and methods

#### Animals

Fifty-four naive male Wistar rats of 9 weeks age were classified into GT, ST, or Int, following the same PCA index score described in experiment 1,

#### Fear conditioning boxes

Six identical Panlab test chambers (LE111 model), each of them 26 cm high, 25 cm long, and 25 cm wide were used for pre-exposition, conditioning, and testing. Each chamber was located inside a soundproof module (LE116 model). The walls of the test chambers were made of acrylic white plastic. The bottom of each chamber had stainless steel rods of 2 mm of diameter spaced at 10mm intervals (center to center). The US lasted for about 1s at non-codified 0.5 mA, 50 Hz AC shock in the legs from a constant current generator (LE100-26 model). A loudspeaker located on the top of the chamber at 70 dB of 2.8 kHz distributed in 25 tones lasting 30s each that were used as conditioned stimulus. The bottom of the chambers was located on top of a platform that recorded the movements of the animal. Experimental software PANLAB Start fear was used to calculate the percentage score of general activity in order to detect the ratio of total movement time.

#### Fear conditioning: Procedure

This phase started a week after the initial procedure in which animals were characterized as ST, GT, or Int. The experimental design was a 3x2 factorial (phenotype as ST, GT, or Int, and exposition to future CS under two conditions; not-exposed (NPe) condition: animals not previously exposed to the CS; exposed condition (Pe): animals exposed to the CS prior to conditioning) ST-NPe/ST-Pe, GT-NP/GT-Pe). For Pe animals, each group received 25 occurrences of an independent tone, while the NPe group was kept for an equivalent period of time in the test chambers with no additional stimuli. The evaluation session for LI started with a 300 s period without stimulation. After that, animals were exposed to 25 trials of a 30 s tone (future CS). The inter-trial interval was 30 ± 10 s. Animals in NPe condition were trained for an equivalent period without CS.

*Conditioning phase*. A single conditioning trial started 30 s after the last tone (or equivalent time period for NPe groups) and consisted of a single pairing of the 30s tone (CS) and an US (electric shock, 1 s, 0,5 mA) at the end of the CS. A 180 s interval separated test from conditioning; during this time, animals underwent trials followed by a single tone for all conditions. General activity was registered during the exposition to the tone (or an equivalent period of time for animals in the NPe condition).

#### Data analysis

As indicated above, ANOVA test and Tukey post-hoc test was used in order to analyze freezing conditioned by groups. Significant level was established at p < .05.

### Results

**Fig 5:**
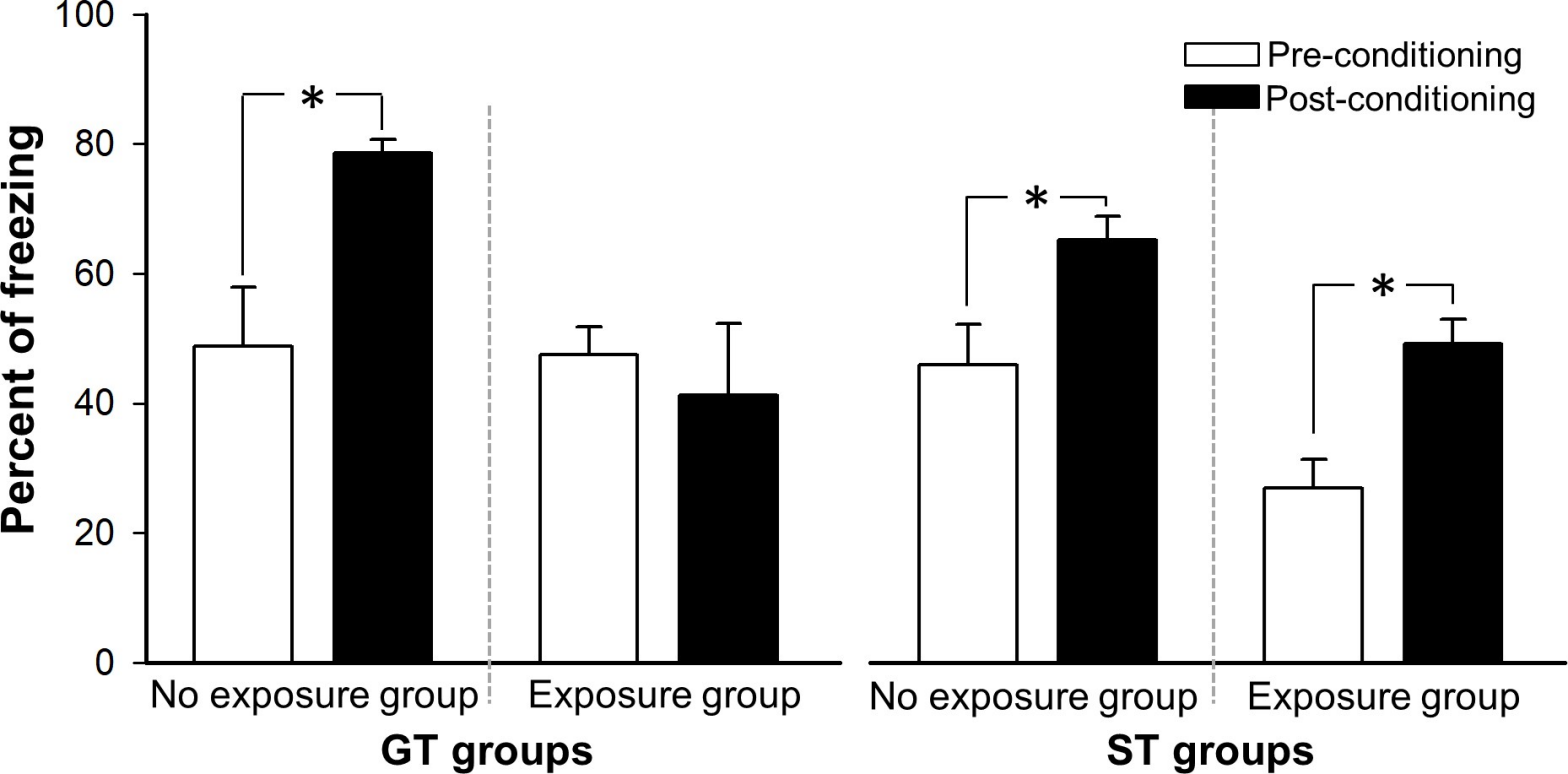
Mean of freezing response in GT and ST groups exposed and non-exposed to the future CS in a LI test. Asterisks indicate the conditioning after CS-US association (p < .05).

Fig 5 shows the performance of both groups in a LI procedure. A mixed ANOVA 2 (Phase: Pre-conditioning and Post-conditioning) x 2 (Preexposure: No exposure group and Exposure group) x 2 Phenotype (GT groups and ST groups) showed a significant effect of Phase variable, F(1, 50) = 12.32, p = 0.001 due to an increased freezing after conditioning phase. The effect of the Preexposure was also significant, F(1, 50) = 20.42, p = 0.000. The interaction Phase x Preexposure x Phenotype was significant, F(1, 50) = 4.42, p = 0.04. The analysis of this interaction showed that during preconditioning phase, animal activity was similar and no statistical differences were found (all ps>0.05). However, during the post-conditioning phase the analysis revealed that in GT Pe group the conditioning was less than the observed in GT NPe group (p = 0.000) and there were no differences between ST-Pe group and ST NPe group (p = 0.54). In the ST conditions there was an effect of Phase, F(1, 26) = 20.33, p = 0.000, and Preexposure, F(1, 26) = 13.04, p = .001 but there was no effect of the interaction Phase x Preexposure (p = 0.74) indicating that for this phenotype rats increased freezing to CS from pre-conditioning to post-conditioning phases but this effect was not modulated by the preexposure. There was a significant difference between preconditioning and post-conditioning phases in ST-Npe group (p = 0.039). Freezing also increased significantly from preconditioning to conditioning phase in ST Pe group (p = 0.018). In the GT conditions, the interaction Phase x Preexposure was significant, F(1, 24) = 4.74, p = 0.04 due to GT NPe rats increased freezing from Pre-conditioinig Phase to Post-conditioning phase (p = 0.002) but GT Pe group displayed a clear LI effect, there was no difference from pre-conditioning phase to conditioning phase (p = 0.519).

## Discussion

We explored the role of the PFC, developmental state, incentive salience of stimuli, and processing of unrewarded stimuli in GT vs ST rats. Lesioning the mPFC affected selectively the ST phenotype. The distribution of animals showing ST and GT phenotypes changed with age, this could be related to developmental trajectory of the mPFC among others developmental factors. Finally, attention to previously exposed stimuli was processed differently in GT vs ST rats.

Experiment 1 data suggest a different role of the mPFC in GT and ST rats. Even though we were not able to know the profile of an animal before lesion, since it would have affected to post-lesion performance, the comparison with a sham allowed us a general sight of the possible involvement of mPFC in ST and GT profile distribution. Although we do not have evidence that both sham and mPFC lesion animals learn the CS-US relationship equally well, lesion to mPFC selectively affected the ST group, reducing the probability of finding a ST profile and leading to an increase in the number of GT animals. This result could be explained by the data obtained by Haight et al. [37], who observed that c-fos activity increased in neurons of the prelimbic cortex after autoshaping training in both GT and ST groups. This increment in activity in the prelimbic cortex correlated with an increment in the activity of the paraventricular nucleus of the thalamus in GT, but not in ST [38]. Haight et al. [37] suggested that greater activity of mPFC neurons could reduce the incentive salience of stimuli, allowing a better top-down control. However, mPFC lesions yielded a reduction in ST, facilitating a GT response. This data is not consistent with a top-down attribution of mPFC but supports the idea of the PFC modulating the attribution of incentive salience to a stimulus. Such control could be accomplished in conjunction with other subcortical areas linked to a cortico-striatal loop that would converge on the NAc [39,40].

Experiment 2 shows the effects of incrementing the incentive salience of a stimulus by increasing the deprivation of food in juvenile vs. late adolescent rats. Immature animals showed a transfer to the ST endophenotype throughout Phase B. It is possible that an immature mPFC does not allow reducing interference processes when animals have to choose between different responses, resulting in high activity of both lever press and number of magazine entries in Phase B. Furthermore, these animals show top-down actions together with the activities typical of an ST endophenotype. On the contrary, late adolescent animals showed a clear change of strategy, moving from a GT to an ST endophenotype when the incentive salience of the CS was increased. However, when they returned to initial conditions they did not show the same performance (Phase A’). This data indicates different profiles could be effective for classification only in the first sessions due to manipulations to the procedure is sensitive to learning processes.

In rodent models, PFC undergoes a marked change in neural circuit connectivity, gene expression, and functionality [31] that could explain the differences found in juvenile vs. late adolescent rats. Previous studies suggest that ST endophenotype can be the expression of a transitory late developmental stage and the persistence of this phenotype in adulthood may be due to genetic factors [41]. The expression of ST endophenotype over GT has been associated to some aspects of impulsive behavior [42], and also with changes in brain neurotransmitters as dopamine or serotonin. The results of Experiment 2 show that there are also motivational factors that affects the expression of these two endophenotypes.

The last experiment analyzed whether exposure to unrewarded external stimuli could yield different outcome of those stimuli when converted to CS in ST vs GT rats. Experiment 3 analyzed the activity of these two groups in one of the most interesting paradigms measuring attentional processing: latent inhibition. While GT animals showed normal conditioning and latent inhibition, the ST group showed a reduced capacity for latent inhibition. These animals showed a greater conditioning than GT animals, indicating a poor ability to reduce attention to irrelevant stimuli. This data is interesting due to the mPFC ability to decrease attention to any salient cue [25]. Other studies using taste aversion conditioning have shown that lesion to the mPFC increased latent inhibition in animals with a limited exposure to future CS but had no effect in animals with extended training. These results have been interpreted as the mPFC could have a role in coordinating controlled and automatic response [25]. In Experiment 3, ST animals showed a reduced latent inhibition with a limited exposure to future CS that could be explain by a failure of attentional processes (habituation?) to irrelevant stimuli. A mPFC dysfunction has been related to increases in attentional levels and therefore to a lowered latent inhibition. For instance, a predisposition to follow cues regarding drugs in ST animals may be a combination of an excessive attribution of incentive properties to the cues, reduced attentional control, and a propensity for impulsive behavior [11,43]. These results are in line with previous data reveling differences in the neural underpinnings of GT and ST behaviors.

In conclusion, using an established model of Pavlovian conditioned approach behavior that categorize the subjects into different phenotypes depending on their behavior, we have shown that the expression of ST and GT phenotypes is modulated by development, motivational state (o incentive salience of the stimuli) and attentional processes. These findings further our understanding of the role of the prefrontal cortex in the expression of these phenotypes to amplify existing variation in behavioral and physiological responses to conditioned cues. Moreover, the differences in GT and ST rats reported here may have implications on mental health risk factors for disorders (see [44]) such as hyperactivity or substance abuse disorder. Our findings could be interpreted as the ST endophenotype presenting traits associated with vulnerability to disorders related to biogenic amines excess activity in the mPFC.

## Supporting information

S1 File Behavioral data from Experiments 1, 2 and 3. (PDF)

## References

[1] Pearce JM, Hall G. A model for Pavlovian learning: variations in the effectiveness of conditioned but not of unconditioned stimuli. Psychol Rev. 1980;87: 532–52. Available: http://www.ncbi.nlm.nih.gov/pubmed/7443916 7443916

[2] Pearce JM. Evaluation and development of a connectionist theory of configural learning [Internet]. Animal Learning and Behavior. Springer-Verlag; 2002 pp. 73–95. 10.3758/BF03192911 12141138

[3] Rescorla R, Wagner A. A theory of Pavlovian conditioning: Variations in the effectiveness of reinforcement and nonreinforcement. Classical conditioning: current research and theory, Vol 2 1972 pp. 64–99. 10.1101/gr.110528.110

[4] Boakes R. Performance on learning to associate a stimulus with positive reinforcement In: Davis H, Hurvitz H, editor. Operant Pavlovian interactions. Hillsdale, NJ: Erlbaum Associate; 1977 pp. 67–97.

[5] Flagel SB, Watson SJ, Robinson TE, Akil H. Individual differences in the propensity to approach signals vs goals promote different adaptations in the dopamine system of rats. Psychopharmacology (Berl). Springer-Verlag; 2007;191: 599–607. 10.1007/s00213-006-0535-8 16972103

[6] Flagel SB, Robinson TE, Clark JJ, Clinton SM, Watson SJ, Seeman P, et al An Animal Model of Genetic Vulnerability to Behavioral Disinhibition and Responsiveness to Reward-Related Cues: Implications for Addiction. Neuropsychopharmacol 2009 352. Nature Publishing Group; 2009;35: 388 10.1038/npp.2009.142 19794408 PMC2794950

[7] Flagel SB, Clark JJ, Robinson TE, Mayo L, Czuj A, Willuhn I, et al A selective role for dopamine in stimulus–reward learning. Nature. Nature Publishing Group; 2011;469: 53–57. 10.1038/nature09588 21150898 PMC3058375

[8] Flagel SB, Waselus M, Clinton SM, Watson SJ, Akil H. Antecedents and consequences of drug abuse in rats selectively bred for high and low response to novelty. Neuropharmacology. Pergamon; 2014;76: 425–436. 10.1016/j.neuropharm.2013.04.033 23639434 PMC3766490

[9] Flagel SB, Robinson TE. Neurobiological basis of individual variation in stimulus-reward learning. Curr Opin Behav Sci. Elsevier; 2017;13: 178–185. 10.1016/j.cobeha.2016.12.004 28670608 PMC5486979

[10] Lopez JC, Karlsson R-M, O’Donnell P. Dopamine D2 Modulation of Sign and Goal Tracking in Rats. Neuropsychopharmacol 2015 409. Nature Publishing Group; 2015;40: 2096 10.1038/npp.2015.68 25759299 PMC4613614

[11] Sarter M, Phillips KB. The Neuroscience of Cognitive-Motivational Styles: Sign- and Goal-Trackers as Animal Models. Behav Neurosci 2018, Vol 132, Pages 1–12. American Psychological Association (APA); 2018;132: 1–12. 10.1037/bne0000226 29355335 PMC5881169

[12] Flagel SB, Chaudhury S, Waselus M, Kelly R, Sewani S, Clinton SM, et al Genetic background and epigenetic modifications in the core of the nucleus accumbens predict addiction-like behavior in a rat model. Proc Natl Acad Sci U S A. National Academy of Sciences; 2016;113: E2861–70. 10.1073/pnas.1520491113 27114539 PMC4878471

[13] O’Donnell P. Adolescent Onset of Cortical Disinhibition in Schizophrenia: Insights From Animal Models. Schizophr Bull. Narnia; 2011;37: 484–492. 10.1093/schbul/sbr028 21505115 PMC3080677

[14] Goto Y, O’Donnell P. Delayed mesolimbic system alteration in a developmental animal model of schizophrenia. J Neurosci. 2002;22: 9070–7. Available: http://www.ncbi.nlm.nih.gov/pubmed/12388614 12388614 10.1523/JNEUROSCI.22-20-09070.2002 PMC6757697

[15] Berridge KC. The debate over dopamine’s role in reward: the case for incentive salience. Psychopharmacology (Berl). 2007;191: 391–431. 10.1007/s00213-006-0578-x 17072591

[16] Berridge KC, Robinson TE. What is the role of dopamine in reward: hedonic impact, reward learning, or incentive salience? Brain Res Rev. Elsevier; 1998;28: 309–369. 10.1016/S0165-0173(98)00019-8 9858756

[17] Goto Y, Grace AA. Limbic and cortical information processing in the nucleus accumbens. Trends Neurosci. Elsevier Current Trends; 2008;31: 552–558. 10.1016/j.tins.2008.08.002 18786735 PMC2884964

[18] O’Donnell P, Grace AA. Synaptic interactions among excitatory afferents to nucleus accumbens neurons: hippocampal gating of prefrontal cortical input. J Neurosci. Society for Neuroscience; 1995;15: 3622–39. 10.1523/JNEUROSCI.15-05-03622.1995 7751934 PMC6578219

[19] Gray J., Moran P., Grigoryan G, Peters S., Young AM., Joseph M. Latent inhibition: the nucleus accumbens connection revisited. Behav Brain Res. Elsevier; 1997;88: 27–34. 10.1016/s0166-4328(97)02313-9 9401705

[20] Groenewegen H, Mulder AB, Beijer AVJ, Wright CI, Wpes FH, Silva DA, et al Hippocampal and amygdaloid interactions in the nucleus accumbens [Internet]. Psychobiology. Springer-Verlag; 1999 10.3758/bf03332111

[21] Haber SN, Fudge JL, McFarland NR. Striatonigrostriatal pathways in primates form an ascending spiral from the shell to the dorsolateral striatum. J Neurosci. Society for Neuroscience; 2000;20: 2369–82. 10.1523/JNEUROSCI.20-06-02369.2000 10704511 PMC6772499

[22] Kunishio K, Haber SN. Primate cingulostriatal projection: Limbic striatal versus sensorimotor striatal input. J Comp Neurol. John Wiley & Sons, Ltd; 1994;350: 337–356. 10.1002/cne.903500302 7533796

[23] Lavín A, Grace AA. Modulation of dorsal thalamic cell activity by the ventral pallidum: Its role in the regulation of thalamocortical activity by the basal ganglia. Synapse. John Wiley & Sons, Ltd; 1994;18: 104–127. 10.1002/syn.890180205 7839311

[24] Saunders BT, Robinson TE. The role of dopamine in the accumbens core in the expression of Pavlovian-conditioned responses. Eur J Neurosci. NIH Public Access; 2012;36: 2521–32. 10.1111/j.1460-9568.2012.08217.x 22780554 PMC3424374

[25] Pérez-Díaz F, Díaz E, Sánchez N, Vargas JP, Pearce JM, López JC. Different involvement of medial prefrontal cortex and dorso-lateral striatum in automatic and controlled processing of a future conditioned stimulus. PLoS One. Public Library of Science; 2017;12: e0189630 10.1371/journal.pone.0189630 29240804 PMC5730208

[26] Homayoun H, Moghaddam B. Differential representation of Pavlovian-instrumental transfer by prefrontal cortex subregions and striatum. Eur J Neurosci. NIH Public Access; 2009;29: 1461–76. 10.1111/j.1460-9568.2009.06679.x 19309320 PMC2871390

[27] Hearst Eliot, Jenkins HM. Sign-tracking: the stimulus-reinforcer relation and directed action [Internet]. Austin Tex.: Psychonomic Society; 1974 Available: https://www.worldcat.org/title/sign-tracking-the-stimulus-reinforcer-relation-and-directed-action/oclc/1895085

[28] Paxinos G, Watson C. The rat brain in stereotaxic coordinates Elsevier; 2007. 10.1016/0165-0270(80)90021-7 6110810

[29] Schoenbaum G, Setlow B, Saddoris MP, Gallagher M. Encoding predicted outcome and acquired value in orbitofrontal cortex during cue sampling depends upon input from basolateral amygdala. Neuron. Elsevier; 2003;39: 855–67. 10.1016/s0896-6273(03)00474-4 12948451

[30] Meyer PJ, Lovic V, Saunders BT, Yager LM, Flagel SB, Morrow JD, et al Quantifying Individual Variation in the Propensity to Attribute Incentive Salience to Reward Cues. PLoS One. Public Library of Science; 2012;7: e38987 10.1371/journal.pone.0038987 22761718 PMC3382216

[31] Caballero A, Tseng KY. GABAergic Function as a Limiting Factor for Prefrontal Maturation during Adolescence. Trends Neurosci. Elsevier Current Trends; 2016;39: 441–448. 10.1016/j.tins.2016.04.010 27233681 PMC4930717

[32] Caballero A, Flores-Barrera E, Cass DK, Tseng KY. Differential regulation of parvalbumin and calretinin interneurons in the prefrontal cortex during adolescence. Brain Struct Funct. NIH Public Access; 2014;219: 395–406. 10.1007/s00429-013-0508-8 23400698 PMC3665762

[33] Berridge KC. Reward learning: Reinforcement, incentives, and expectations. Psychol Learn Motiv. Academic Press; 2000;40: 223–278. 10.1016/S0079-7421(00)80022-5

[34] Toates FM (Frederick M. Motivational systems [Internet]. Cambridge: Cambridge University Press; 1986 Available: https://www.worldcat.org/title/motivational-systems/oclc/924949860

[35] Franken IHA, Booij J, van den Brink W. The role of dopamine in human addiction: From reward to motivated attention. Eur J Pharmacol. Elsevier; 2005;526: 199–206. 10.1016/j.ejphar.2005.09.025 16256105

[36] Robinson TE, Berridge KC. The neural basis of drug craving: An incentive-sensitization theory of addiction. Brain Res Rev. Elsevier; 1993;18: 247–291. 10.1016/0165-0173(93)90013-P 8401595

[37] Haight JL, Fuller ZL, Fraser KM, Flagel SB. A food-predictive cue attributed with incentive salience engages subcortical afferents and efferents of the paraventricular nucleus of the thalamus. Neuroscience. Pergamon; 2017;340: 135–152. 10.1016/j.neuroscience.2016.10.043 27793779 PMC5154807

[38] Haight JL, Flagel SB. A potential role for the paraventricular nucleus of the thalamus in mediating individual variation in Pavlovian conditioned responses. Front Behav Neurosci. Frontiers Media SA; 2014;8: 79 10.3389/fnbeh.2014.00079 24672443 PMC3953953

[39] Belin D, Balado E, Piazza PV, Deroche-Gamonet V. Pattern of Intake and Drug Craving Predict the Development of Cocaine Addiction-like Behavior in Rats. Biol Psychiatry. Elsevier; 2009;65: 863–868. 10.1016/j.biopsych.2008.05.031 18639867

[40] Haber SN. Corticostriatal circuitry. Dialogues Clin Neurosci. Les Laboratoires Servier; 2016;18: 7–21. Available: http://www.ncbi.nlm.nih.gov/pubmed/27069376 27069376 10.31887/DCNS.2016.18.1/shaber PMC4826773

[41] Campus P, Accoto A, Maiolati M, Latagliata C, Orsini C. Role of prefrontal 5-HT in the strain-dependent variation in sign-tracking behavior of C57BL/6 and DBA/2 mice. Psychopharmacology (Berl). Springer Berlin Heidelberg; 2016;233: 1157–1169. 10.1007/s00213-015-4192-7 26728892

[42] Tomie A, Grimes KL, Pohorecky LA. Behavioral characteristics and neurobiological substrates shared by Pavlovian sign-tracking and drug abuse. Brain Res Rev. NIH Public Access; 2008;58: 121–35. 10.1016/j.brainresrev.2007.12.003 18234349 PMC2582385

[43] Pitchers KK, Sarter M, Robinson TE. The hot “n” cold of cue-induced drug relapse. Learn Mem. Cold Spring Harbor Laboratory Press; 2018;25: 474–480. 10.1101/lm.046995.117 30115769 PMC6097766

[44] Vargas JP, Díaz E, Portavella M, López JC. Animal Models of Maladaptive Traits: Disorders in Sensorimotor Gating and Attentional Quantifiable Responses as Possible Endophenotypes. Front Psychol. Frontiers; 2016;7: 206 10.3389/fpsyg.2016.00206 26925020 PMC4759263

