# Supplementary material for: Sign and goal tracker rats process differently the incentive salience of a conditioned stimulus
Source: PLoS One. 2019 Sep 30;14(9):e0223109. doi: 10.1371/journal.pone.0223109 (PMC6768469; doi:10.1371/journal.pone.0223109)
Supplement: S1 File — (PDF) [file pone.0223109.s001.pdf]

Data of Experiment 1

Subject	Lesion	PCA 1	PCA 2	PCA 3	PCA 4
P01	mPfc	0,1	-0,5	0,8	0,6
P02	mPfc	0,1	0,1	-0,6	-0,4
P03	mPfc	-0,2	0,0	0,0	-0,9
P04	mPfc	0,0	0,0	-0,7	-0,9
P05	mPfc	-0,5	-0,3	-0,8	-0,7
P06	mPfc	0,0	0,0	0,0	0,3
P07	mPfc	0,7	0,4	-0,2	-0,5
P08	mPfc	0,5	0,0	0,1	0,2
P09	mPfc	1,0	-0,3	-0,6	-0,1
P10	mPfc	0,0	0,0	0,0	0,0
P11	mPfc	0,0	0,3	-0,9	-0,9
P12	mPfc	-0,1	-0,1	-1,0	-0,7
P13	mPfc	-0,3	-0,8	-0,7	-0,9
P14	mPfc	-0,8	-1,0	-1,0	0,0
P15	mPfc	0,0	0,0	0,0	0,0
P16	mPfc	1,0	-0,6	-1,0	-0,9
P17	mPfc	0,5	0,5	-0,9	-0,8
P18	mPfc	0,2	0,0	-0,7	-0,6
P19	mPfc	1,0	1,0	0,1	-0,7
P20	mPfc	1,0	0,0	-1,0	-1,0
P21	mPfc	0,0	-0,5	-0,2	-0,2
P22	mPfc	-0,7	-0,6	-1,0	-0,7
P23	mPfc	-1,0	0,0	-1,0	-1,0
P24	mPfc	1,0	0,2	0,0	-0,1
P25	mPfc	-0,8	0,0	-1,0	-1,0
S01	Sham	-1,0	-1,0	-0,3	-0,2
S02	Sham	-1,0	-0,4	-0,5	-0,9
S03	Sham	-0,2	-0,9	-0,2	-0,9
S04	Sham	1,0	-0,2	-0,4	-0,3
S05	Sham	0,6	-0,6	-0,1	-0,6
S06	Sham	0,3	0,4	-0,2	-0,2
S07	Sham	0,0	-0,8	0,1	-0,4
S08	Sham	0,5	0,9	0,8	0,9
S09	Sham	-1,0	-0,7	0,8	0,6
S10	Sham	0,9	1,0	0,9	0,9
S11	Sham	0,0	0,3	0,5	0,3
S12	Sham	-0,6	0,2	0,5	0,6
S13	Sham	-1,0	-0,7	0,5	0,0
S14	Sham	-1,0	-0,3	0,7	0,8
S15	Sham	-0,2	-0,1	0,7	0,5
S16	Sham	-0,7	-0,6	-0,2	-0,3
S17	Sham	0,9	0,0	-0,1	0,3
S18	Sham	0,3	-0,8	-0,5	-0,7
S19	Sham	-0,8	-0,5	-0,5	-0,4
S20	Sham	-0,9	-0,4	-0,4	-0,5
S21	Sham	-1,0	-0,7	-0,4	-0,8
S22	Sham	-1,0	-0,9	-0,3	-0,1
S23	Sham	0,6	0,8	1,0	0,8

S24	Sham	0,8	0,9	1,0	0,8
S25	Sham	0,9	0,7	1,0	1,0

Data of Experiment 2

	Phase A				Phase B
**Group**	**PCA 1**	**PCA 2**	**PCA 3**	**PCA 4**	**PCA 1**
pre-adolescent	1,0	-0,7	-0,6	-0,7	-0,5
pre-adolescent	-0,8	-0,4	-1,0	-1,0	-0,9
pre-adolescent	-0,6	-0,4	-0,1	-0,1	-0,3
pre-adolescent	-0,7	-0,8	-0,6	-0,7	-0,4
pre-adolescent	-0,7	0,1	0,5	0,9	1,0
pre-adolescent	0,4	0,4	0,4	0,7	0,7
pre-adolescent	1,0	-0,2	1,0	0,4	-0,4
pre-adolescent	-0,1	0,8	1,0	0,7	0,9
pre-adolescent	-0,6	-0,2	-0,1	0,4	0,5
pre-adolescent	-0,4	-0,7	0,1	-0,2	0,3
pre-adolescent	0,0	-0,6	0,1	-0,3	0,2
pre-adolescent	-0,3	0,7	0,1	0,0	0,7
pre-adolescent	-0,7	-0,7	-0,6	-0,6	-0,7
pre-adolescent	0,2	-0,9	-1,0	-0,6	0,1
pre-adolescent	-0,9	-0,5	-0,7	-0,8	-0,8
pre-adolescent	0,9	1,0	0,4	1,0	0,8
pre-adolescent	-0,3	-0,8	0,5	0,8	0,8
pre-adolescent	1,0	0,6	1,0	1,0	1,0
pre-adolescent	0,8	1,0	0,9	1,0	1,0
pre-adolescent	0,0	0,5	1,0	0,9	1,0
pre-adolescent	0,3	0,2	1,0	0,7	0,5
pre-adolescent	-1,0	-0,6	1,0	0,9	0,7
pre-adolescent	0,0	0,4	0,6	0,8	0,7
pre-adolescent	-0,9	0,1	0,8	0,9	0,8
pre-adolescent	1,0	1,0	0,2	1,0	-1,0
pre-adolescent	1,0	0,2	0,9	1,0	-0,9
pre-adolescent	0,4	-0,2	-0,7	-0,5	-0,6
pre-adolescent	0,1	0,6	0,8	0,1	-0,3
pre-adolescent	-0,9	0,1	0,2	0,2	-0,3
pre-adolescent	0,1	-0,4	-0,5	0,3	-0,1
pre-adolescent	-0,2	-0,5	1,0	-0,2	0,0
pre-adolescent	-0,1	0,2	1,0	0,3	0,9
pre-adolescent	0,9	0,5	0,1	-0,5	0,7
Late adolescent	-1,0	-0,4	-0,9	-0,5	-0,8
Late adolescent	-0,2	-0,9	-0,9	-0,2	1,0
Late adolescent	0,6	-0,6	-0,6	-0,1	0,5
Late adolescent	0,3	-0,8	-0,7	-0,5	0,4
Late adolescent	-0,8	-0,5	-0,4	-0,5	-0,2
Late adolescent	-1,0	-0,7	-0,8	-0,4	0,5
Late adolescent	-0,4	0,0	0,0	-1,0	-0,2
Late adolescent	0,5	0,9	0,9	0,8	0,5
Late adolescent	-1,0	-0,7	0,6	0,8	0,7
Late adolescent	0,9	1,0	0,9	0,9	0,9
Late adolescent	0,0	0,3	0,3	0,5	0,9

Late adolescent	-1,0	-0,7	0,0	0,5	0,7
Late adolescent	-1,0	-0,3	0,8	0,7	0,8
Late adolescent	-0,2	-0,1	0,5	0,7	0,4
Late adolescent	0,6	0,8	0,8	1,0	0,9
Late adolescent	0,8	0,9	0,8	1,0	0,9
Late adolescent	0,9	0,6	1,0	1,0	1,0
Late adolescent	-0,8	0,5	0,9	1,0	1,0
Late adolescent	0,4	0,6	0,9	1,0	0,8
Late adolescent	0,4	0,6	1,0	0,9	0,9
Late adolescent	0,3	0,8	0,4	1,0	0,2
Late adolescent	0,2	1,0	0,7	1,0	1,0
Late adolescent	0,1	0,8	0,7	0,9	0,9
Late adolescent	0,7	0,8	1,0	0,6	1,0
Late adolescent	-0,5	0,9	0,1	1,0	0,7
Late adolescent	-1,0	-1,0		0,6	0,9
Late adolescent	-1,0	-1,0	-0,2	-0,3	0,0
Late adolescent	1,0	-0,2	-0,3	-0,4	0,8
Late adolescent	0,3	0,4	-0,2	-0,2	0,3
Late adolescent	0,0	-0,8	-0,4	0,1	0,5
Late adolescent	-0,6	0,2	0,6	0,5	0,5
Late adolescent	-0,7	-0,6	-0,3	-0,2	0,8
Late adolescent	0,9	0,0	0,3	-0,1	0,4
Late adolescent	-0,9	-0,4	-0,5	-0,4	-0,4
Late adolescent	-1,0	-0,9	-0,1	-0,3	0,0
Late adolescent	0,6	0,3	0,5	0,4	0,5
Late adolescent	0,9	-1,0	-0,9	-0,1	0,5
Late adolescent	0,0	0,1	0,5	0,5	0,3
Late adolescent	0,9	0,7	0,7	0,3	0,8

			Phase A'			
PCA 2	PCA 3	PCA 4	PCA 1	PCA 2	PCA 3	PCA 4
-0,7	0,9	1,0	0,9	1,0	1,0	1,0
-1,0	-1,0	-0,6	-0,6	-0,3	-0,1	-0,5
0,9	1,0	1,0	0,9	1,0	1,0	1,0
-0,6	0,9	0,9	0,7	0,7	0,9	0,7
0,9	0,9	0,9	0,9	0,9	0,7	0,7
1,0	1,0	1,0	1,0	1,0	1,0	1,0
0,6	1,0	1,0	0,9	1,0	1,0	1,0
1,0	1,0	1,0	1,0	1,0	0,9	0,9
0,7	0,7	0,4	0,4	0,7	0,7	0,5
1,0	1,0	1,0	1,0	1,0	1,0	1,0
0,9	1,0	1,0	1,0	1,0	1,0	1,0
0,9	1,0	1,0	0,9	1,0	1,0	0,7
-0,7	-0,9	-0,8	-0,6	-0,4	-0,4	-0,3
0,2	0,4	0,4	0,0	0,3	0,2	0,3
-0,2	0,3	0,9	0,7	0,5	0,6	0,4
0,9	1,0	1,0	1,0	1,0	1,0	1,0
1,0	1,0	1,0	1,0	1,0	1,0	1,0
1,0	1,0	1,0	1,0	1,0	1,0	0,9
1,0	0,9	1,0	1,0	1,0	1,0	1,0
1,0	0,9	1,0	1,0	0,9	1,0	0,9
0,5	0,2	0,2	0,8	0,0	-0,1	-0,1
0,8	0,5	0,8	1,0	1,0	0,9	1,0
0,9	1,0	1,0	1,0	1,0	1,0	0,9
1,0	1,0	1,0	1,0	1,0	1,0	1,0
-1,0	-0,7	1,0	0,4	1,0	0,6	0,9
-1,0	-1,0	-1,0	-0,9	-0,9	-0,8	-1,0
0,6	1,0	1,0	0,6	1,0	1,0	1,0
-0,2	0,8	0,4	1,0	1,0	1,0	1,0
0,2	0,8	1,0	0,5	0,7	0,7	0,7
0,9	1,0	1,0	1,0	0,9	1,0	0,9
0,8	0,9	1,0	0,8	0,9	0,9	0,9
0,9	0,9	0,9	0,8	0,9	1,0	1,0
0,2	0,5	0,9	0,9	0,9	0,9	0,5
0,3	0,9	1,0	1,0	1,0	1,0	1,0
1,0	1,0	1,0	0,9	1,0	1,0	0,9
0,7	0,8	0,9	0,9	0,9	1,0	0,9
0,8	0,8	0,4	0,7	0,5	0,7	0,8
0,3	0,6	0,1	0,2	-0,1	-0,3	-0,2
0,6	0,9	1,0	0,5	0,7	0,9	0,9
0,8	1,0	1,0	1,0	1,0	1,0	1,0
0,8	0,9	0,7	0,8	0,9	0,7	0,5
1,0	0,9	1,0	1,0	0,8	0,9	1,0
1,0	1,0	1,0	0,9	1,0	1,0	1,0
0,7	0,9	1,0	1,0	1,0	1,0	1,0

1,0	1,0	0,9	1,0	1,0	0,9	0,9
1,0	1,0	1,0	0,9	1,0	0,9	0,9
0,2	0,5	0,8	0,7	0,9	0,8	1,0
1,0	1,0	1,0	1,0	0,9	1,0	1,0
0,9	1,0	1,0	1,0	1,0	1,0	1,0
1,0	0,9	1,0	0,9	1,0	1,0	1,0
1,0	1,0	1,0	1,0	1,0	0,9	0,8
1,0	1,0	1,0	1,0	1,0	1,0	1,0
1,0	1,0	1,0	0,9	0,9	1,0	1,0
0,8	1,0	1,0	1,0	1,0	1,0	1,0
1,0	1,0	1,0	0,9	1,0	0,9	0,8
1,0	0,9	1,0	1,0	1,0	0,9	0,9
1,0	1,0	1,0	1,0	1,0	1,0	1,0
0,9	-0,7	0,9	0,9	0,7	0,8	1,0
1,0	1,0	1,0	1,0	0,9	1,0	1,0
0,2	0,0	0,6	0,9	0,9	1,0	0,8
1,0	1,0	1,0	0,2	1,0	1,0	1,0
0,5	0,5	0,5	1,0	0,5	0,3	0,9
0,9	1,0	1,0	1,0	1,0	1,0	1,0
0,8	1,0	1,0	1,0	1,0	1,0	1,0
1,0	1,0	1,0	0,8	1,0	1,0	1,0
0,8	0,9	0,9	0,1	0,6	0,7	0,8
-0,2	0,1	0,2	1,0	-0,1	-0,4	-0,4
0,8	0,9	1,0	0,9	0,9	1,0	1,0
0,9	1,0	1,0	0,8	0,9	1,0	1,0
0,9	1,0	1,0	0,3	0,9	0,9	1,0
0,6	-0,1	-0,3	1,0	0,2	0,1	-0,2
1,0	1,0	1,0		1,0	0,9	0,9

Data of Experiment 3

		% Freezing	
**Phenotype**	**Preexposure**	**before conditioning**	**after conditioning**
Goal Tracker	No preexposed	95,6	87,3
Goal Tracker	No preexposed	92,8	82,9
Goal Tracker	No preexposed	91,4	82,8
Goal Tracker	No preexposed	89,3	85,9
Goal Tracker	No preexposed	65,3	70,5
Goal Tracker	No preexposed	60,2	68,4
Goal Tracker	No preexposed	51,4	74,2
Goal Tracker	No preexposed	48,9	68,3
Goal Tracker	No preexposed	22,2	79,4
Goal Tracker	No preexposed	21,1	91,2
Goal Tracker	No preexposed	20,3	88,4
Goal Tracker	No preexposed	18,1	78,4
Goal Tracker	No preexposed	7,3	68,8
Goal Tracker	No preexposed	0,5	74,0
Goal Tracker	Exposed	66,8	13,5
Goal Tracker	Exposed	64,3	11,6
Goal Tracker	Exposed	61,4	20,1
Goal Tracker	Exposed	57,9	12,5
Goal Tracker	Exposed	49,7	6,2
Goal Tracker	Exposed	49,5	92,5
Goal Tracker	Exposed	47,4	6,1
Goal Tracker	Exposed	44,7	88,4
Goal Tracker	Exposed	44,0	26,1
Goal Tracker	Exposed	43,9	30,1
Goal Tracker	Exposed	22,3	92,1
Goal Tracker	Exposed	18,9	96,9
Sign Tracker	No preexposed	64,6	91,9
Sign Tracker	No preexposed	88,2	62,7
Sign Tracker	No preexposed	67,4	70,0
Sign Tracker	No preexposed	62,4	66,4
Sign Tracker	No preexposed	60,3	68,1
Sign Tracker	No preexposed	57,7	58,4
Sign Tracker	No preexposed	51,8	74,0
Sign Tracker	No preexposed	30,8	50,9
Sign Tracker	No preexposed	49,6	69,4
Sign Tracker	No preexposed	43,6	32,2
Sign Tracker	No preexposed	27,6	62,9
Sign Tracker	No preexposed	22,3	55,1
Sign Tracker	No preexposed	11,6	74,8
Sign Tracker	No preexposed	4,7	75,5
Sign Tracker	Exposed	55,5	60,8
Sign Tracker	Exposed	49,6	55,9
Sign Tracker	Exposed	22,2	45,4
Sign Tracker	Exposed	39,0	57,2

Sign Tracker	Exposed	37,1	25,1
Sign Tracker	Exposed	36,6	52,9
Sign Tracker	Exposed	35,1	54,1
Sign Tracker	Exposed	29,7	49,1
Sign Tracker	Exposed	27,5	36,5
Sign Tracker	Exposed	25,8	32,6
Sign Tracker	Exposed	8,2	70,2
Sign Tracker	Exposed	7,3	38,9
Sign Tracker	Exposed	3,8	74,8
Sign Tracker	Exposed	0,4	36,6
